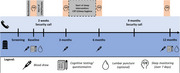# Double blind clinical trial of lemborexant for AD prevention: Protocol for a randomized controlled clinical trial

**DOI:** 10.1002/alz70859_105733

**Published:** 2025-12-26

**Authors:** Nolan‐Patrick Cunningham, Jennifer Tremblay‐Mercier, Andrée‐Ann Baril, Thien Thanh Dang‐Vu, Andrew Lim, Maiya R. Geddes, Mohamed Badawy, Judes Poirier, Simon Ducharme, Sylvia Villeneuve, for the PREVENT‐AD Research Group

**Affiliations:** ^1^ Douglas Mental Health University Institute, Montreal, QC Canada; ^2^ Centre for Studies on Prevention of Alzheimer’s Disease (StoP‐AD Centre), Montreal, QC Canada; ^3^ Center for Advanced Research in Sleep Medicine, Hôpital du Sacré‐Coeur de Montréal, CIUSSS‐NIM, Montreal, QC Canada; ^4^ Université de Montréal, Montreal, QC Canada; ^5^ Centre de recherche de l'Institut universitaire de gériatrie de Montréal, Montreal, QC Canada; ^6^ Concordia University, Montreal, QC Canada; ^7^ University of Toronto, Toronto, ON Canada; ^8^ Montreal Neurological Institute, McGill University, Montreal, QC Canada; ^9^ McGill University, Montreal, QC Canada

## Abstract

**Background:**

Poor sleep is a modifiable factor associated with the development and progression of Alzheimer’s Disease (AD). Pharmacological treatments and Cognitive Behavioural Therapy for Insomnia (CBT‐I) which promote sleep have the potential to improve AD risk profile. In addition, prior research suggests that Dual Orexin Receptor Antagonists (DORAs), which are prescribed more and more to treat insomnia, could also help to decrease tau phosphorylation and concentrations of beta‐amyloid (Aβ) in the cerebrospinal fluid.

**Objective:**

To study the impact of Lemborexant, a DORA, with or without CBT‐I on AD blood biomarkers and on cognitive performance in a population at risk of developing AD dementia.

**Method:**

We will conduct a double‐blind randomized clinical trial in men and women aged 50 to 90 years old with symptoms of insomnia. 220 participants will be screened and randomized into 4 treatment groups over 12 months: Lemborexant, Lemborexant plus CBT‐I, placebo, or placebo plus CBT‐I. All participants will receive education on sleep hygiene measures.

**Primary Outcomes:**

Change in (1) plasma p‐tau181 and (2) modified Preclinical Alzheimer’s Cognitive Composite score from baseline to 12‐months.

**Secondary Outcomes:**

Change in (1) plasma p‐tau217, (2) CSF Aβ 42/40 and CSF p‐tau181, and (3) objective sleep measures measured with electroencephalogram (EEG) recording from baseline to 12‐months.

**Anticipated Results:**

We anticipate that Lemborexant will be associated with a reduction in plasma p‐tau181 over time compared to placebo. Lemborexant will improve sleep and cognitive performance compared to placebo, and the addition of CBT‐I will accentuate the beneficial effect on sleep and cognition but not on AD biomarkers.

**Conclusion:**

This study will grant us an opportunity to deepen our understanding of the therapeutic potential of sleep interventions and the influence of DORAs on AD prevention.